# Associations Between Left DLPFC iTBS-induced Functional Connectivity Changes and Depressive Symptoms: An Exploratory Study

**DOI:** 10.62641/aep.v53i6.1983

**Published:** 2025-12-17

**Authors:** Kun Xie, Wenshuang Yang, Chengfeng Chen, Shiqi Yuan, Zeyang Zhao, Shiying Wang, Jiang Wang, Peiying Li, Bin Zhang

**Affiliations:** ^1^Department of Psychiatry, The Affiliated Brain Hospital of Guangzhou Medical University, 510370 Guangzhou, Guangdong, China; ^2^Department of Psychiatry, Guangzhou Medical University, 510180 Guangzhou, Guangdong, China; ^3^Department of Psychiatry, Tianjin Anding Hospital, Mental Health Center of Tianjin Medical University, 300222 Tianjin, China; ^4^Department of Psychology, Chengde Medical University, 067000 Chengde, Hebei, China; ^5^Department of Psychiatry and Psychology, Tianjin Medical University, 301700 Tianjin, China; ^6^Mental Health Center of Tianjin University, Tianjin Anding Hospital, 300222 Tianjin, China; ^7^Institute of Mental Health, Tianjin Anding Hospital, Mental Health Center of Tianjin Medical University, 300222 Tianjin, China

**Keywords:** major depressive disorder, intermittent theta-burst stimulation, functional connectivity

## Abstract

**Background:**

As a more efficient variant of repetitive transcranial magnetic stimulation (rTMS), intermittent theta-burst stimulation (iTBS) has been shown to effectively treat major depressive disorder (MDD). Resting-state functional connectivity (FC) is believed to help explain how iTBS exerts its therapeutic effects. Research findings regarding FC changes induced by iTBS targeting the left dorsolateral prefrontal cortex (DLPFC) are inconsistent, warranting exploratory investigations. In this study, we analyzed the effects of a 10-day iTBS treatment on changes in resting-state FC in patients with MDD.

**Methods:**

This study enrolled 29 patients with MDD from Tianjin Anding Hospital between February 2023 and November 2024. These patients received 10 days of left DLPFC iTBS treatment, with its efficacy closely monitored. A FC matrix was constructed using the Human Brainnetome Atlas as a template. Changes in FC were analyzed, and correlation analysis was conducted between baseline FC in different brain regions and depressive symptoms. Regression analysis was then performed to predict depressive symptoms improvement based on baseline FC.

**Results:**

Our results demonstrate that iTBS treatment yields a significant therapeutic effect, with response and remission rates of 62.07% and 31.03%, respectively. FC analysis revealed a reduction in positive FC between the ventromedial putamen (vmPu), precentral gyrus (PrG), and postcentral gyrus (PoG). The correlation between baseline FC of vmPu and PrG (r = 0.529, *p* = 0.003), as well as between vmPu and PoG (r = 0.545, *p* = 0.002), was found to be positively associated with improvements in depressive symptoms. Additionally, linear regression analysis indicated that these baseline FC predicted the extent of therapeutic improvement.

**Conclusion:**

Intervention with iTBS has demonstrated promising therapeutic effects in the treatment of depression. This study identified FC that correlates with treatment response and could predict improvement in depressive core symptoms.

**Trial Registration:**

Chinese Clinical Trial Registry (ChiCTR2100054793).

## Introduction

Major Depressive Disorder (MDD) is a prevalent mental illness and one of the leading causes of disability, posing a significant public health challenge [1]. A mental health survey conducted in China found that the annual prevalence of depression is 3.6%, while the lifetime prevalence reaches 6.9% [2]. However, the treatment rate for depression remains low, with few individuals receiving adequate care [3]. Currently, antidepressants are the primary treatment for MDD, but they are often accompanied by issues such as low remission rates, delayed onset of effects, and common side effects [4, 5], some of which may negatively impact treatment adherence [6].

As a non-invasive neuromodulation technique, repetitive transcranial magnetic stimulation (rTMS) is known for its high safety profile and low risk of side effects [7]. In 2018, rTMS targeting the left dorsolateral prefrontal cortex (DLPFC) was approved by the U.S. Food and Drug Administration (FDA) for the treatment of treatment-resistant depression [8]. As a more efficient variant of rTMS [9], intermittent theta-burst stimulation (iTBS) has garnered increasing attention in recent years due to its higher pulse density and shorter treatment duration. It has also been approved by the FDA for the treatment of treatment-resistant depression. Research indicates that iTBS achieves a response rate of 49% and a remission rate of 32% in patients with depression [8]. The study of Stanford Neuromodulation Therapy (SNT) even reported a response (Montgomery–Asberg Depression Rating Scale (MADRS) score decreased by 50%) rate of 85.7% and a remission (a MADRS score ≤10) rate of 78.6% at the 4-week follow-up [10], highlighting the significant role of accelerated iTBS in the treatment of MDD. These findings underscore the need for further investigation into its efficacy and underlying mechanism.

ITBS generates induced currents by applying magnetic pulses to DLPFC, modulating its activation or inhibition, which results in widespread functional changes across brain regions, including alterations in functional connectivity (FC). These changes are likely closely associated with the antidepressant effects of iTBS. Baeken *et al*’s randomized clinical trial [11] suggests that the FC of subgenual anterior cingulate cortex can distinguish responders from non-responders to iTBS treatment. Another study [12] proposed increased FC between the dorsomedial prefrontal cortex and regions overlapping the precuneus and posterior cingulate cortex after active treatment compared with sham treatment. In addition, FC between the precuneus and therapeutic targets predicted symptom improvement [12]. However, Struckmann* et al*’s study [13] found decreased DLPFC FC to the right insula compared with sham group and found their correlation with an improvement in symptoms. These studies listed above, which were individually able to predict symptoms, were similar in design but produced different FC results. This inconsistency prompted investigation into relationships between FC of brain regions and antidepressant efficacy, and whether FC can predict the treatment outcome.

For this exploratory study, we will recruit participants with MDD to investigate changes in FC between different brain regions following iTBS treatment and their correlations with antidepressant efficacy. Participants will undergo a 10-day DLPFC iTBS intervention, as well as resting-state functional magnetic resonance imaging (fMRI) scans and assessments before and after the intervention. FCanalysis will then be conducted. We anticipate identifying specific FC patterns that are strongly correlated with improvements in depressive symptoms and could be used to predict antidepressant efficacy based on baseline values.

## Materials and Methods

### Participants

All participants were recruited from the outpatient department of Tianjin Anding Hospital between February 2023 and November 2024. Each participant completed a 10-day intervention protocol with associated data collection according to the study design. Prior to enrollment, all participants were informed about and fully understood the details of the experiment and potential risks. They agreed to participate in the study, which involved magnetic resonance imaging (MRI), iTBS intervention, and neuropsychological assessments. After being informed of the research plan, all participants voluntarily signed an informed consent form. All procedures of this study were approved by the Ethics Committee of Tianjin Anding Hospital (Ethics approval number (2021) No. 040 and (2022) No. 2022-43).

The inclusion criteria for this study are as follows: (1) Age between 18 and 60 years; (2) Diagnosis of MDD according to the Diagnostic and Statistical Manual of Mental Disorders, Fifth Edition (DSM-5) [14], and confirmed by the Mini International Neuropsychiatric Interview (M.I.N.I.); (3) The 17-item Hamilton Depression Rating Scale (HAMD) [15] score greater than or equal to 17.

The exclusion criteria are as follows: (1) Severe organic diseases, such as epilepsy, stroke, brain injury, or serious physical illnesses, such as malignant tumors, multi-organ failure; (2) Co-occurring psychiatric disorders, such as bipolar disorder, schizophrenia; (3) Contraindications for MRI or transcranial magnetic stimulation (TMS), such as those with claustrophobia or those with metal implants in their bodies; (4) Received electroconvulsive therapy (ECT), TMS, or light therapy within the past 6 months; (5) History of alcohol or drug abuse within the past year; (6) Pregnant women.

The dropout criteria are as follows: (1) Missing treatment for more than four consecutive days; (2) Voluntary withdrawal of informed consent; (3) Experiencing intolerable adverse reactions or severe adverse events; (4) Development of manic or psychotic symptoms.

### Clinical Assessments

All participants’ severity of depressive symptoms is assessed using the 17-item Hamilton Depression Rating Scale at baseline and within 24 hours after the last session of transcranial magnetic stimulation treatment. Based on Romera *et al*. [16], HAMD factor scores are defined. The retardation factor is defined as the sum of items 1, 7, 8, and 14 of the HAMD scale, the cognitive impairment factor is defined as the sum of items 2, 3, and 9, the anxiety factor was defined as the sum of items 10, 11, 12, 13, 15, and 17, and the sleep disturbance factor is defined as the sum of items 4, 5, and 6. Moreover, the reduction rate is defined as the difference between the pre-treatment and post-treatment scale scores, divided by the pre-treatment scale score. The response rate is defined as the proportion of participants whose HAMD score decreases by more than 50% after receiving iTBS treatment, while the remission rate is defined as the proportion of participants with a HAMD score of 8 or below after receiving iTBS treatment.

### iTBS Procedure

This study used the MagVenture transcranial magnetic stimulation device (MagPro X100 incl MagOption, MagVenture, Farum, Denmark) with a figure-eight coil for stimulation. Prior to stimulation, the Northern Digital Inc. navigator (Polaris Vicra, Northern Digital Inc., Waterloo, Canada) was used to locate the stimulation target, which was selected from the left DLPFC defined as the combined extent of 20 mm radius spheres centered along the left hemisphere at four TMS stimulation sites [17]. Before the first treatment, the physician measured the individual stimulus intensity according to the resting motor threshold (RMT), which is defined as the minimum stimulus intensity that generates a motor-evoked potential of greater than 50 µV in 50% of 10 times. The iTBS treatment protocol for the entire study consisted of 20 sessions, with two sessions per day and a minimum interval of 50 minutes between each session, completed over 10 consecutive days. The specific iTBS parameters were as follows: the stimulation intensity was set at 100% of the resting motor threshold, with the option to adjust the intensity based on participant feedback. Each session consisted of 100 trains of stimulation, with each train lasting two seconds of stimulation followed by eight seconds of rest. Each train comprised 10 bursts at 5 Hz, and each burst included three biphasic pulses at 50 Hz. Thus, a total of 3000 pulses were delivered per session.

### MRI Acquisition and fMRI Preprocessing

The MRI data, including T1 and resting-state functional MRI (rs-fMRI), were acquired with a 3.0-Tesla magnetic resonance apparatus (MAGNETOM Prisma, Siemens, Munich, Germany) located at Tianjin Anding Hospital. The T1 scan was acquired using a magnetization prepared rapid acquisition gradient echoes (MPRAGE) sequence, and parameters were set as follow: repetition time = 2000 ms; echo time = 2.32 ms; voxel size = 0.9 mm × 0.9 mm × 0.9 mm; slice thickness = 0.9 mm; flip angle = 8 deg; generalized autocalibrating partially parallel acquisition (GRAPPA) = 2; Field of view (FOV) = 230 mm × 230 mm; matrix = 256 × 256; 208 slices. All the participants were instructed to keep still, close their eyes, and relax while remaining awake. A gradient-recalled echo-planar imaging (EPI) sequence was used as follows: repetition time = 800 ms; echo time = 30 ms; voxel size = 2 mm × 2 mm × 2 mm; slice thickness = 2 mm; flip angle = 56 deg; FOV = 208 mm × 208 mm; matrix = 104 × 104; 72 slices. A total of 450 volumes were collected and the scanning time was 374 s.

All fMRI data were preprocessed using the Data Processing Assistant for Resting-State (DPARSFA) fMRI Advanced Edition (DPARSFA v4.5, Chao-Gan Yan, Beijing, China) software of the Data Processing and Analysis of Brain Imaging (DPABI, Chao-Gan Yan, Beijing, China) toolkit (http://rfmri.org/dpabi) [18]. In brief, the preprocessing steps were as follow: Discarding the first 10 volumes; Head motion correction by realignment, and the data were excluded where translational or rotational motion parameters exceeded ±3.0 mm or ±3.0°. Spatial normalization using the standard EPI Montreal Neurological Institute (MNI) template at a resolution of 3 × 3 × 3 mm^3^, and linear detrending and temporal bandpass filtering (0.01~0.1 Hz). Several nuisance covariates, including 24 motion parameters and averaged signals from the white matter and cerebrospinal fluid, were regressed out.

### Resting-state FCAnalysis

To explore the changes in FC across brain regions before and after iTBS treatment, this study used the Human Brainnetome Atlas (https://atlas.brainnetome.org) [19] as a template for brain region segmentation. The whole brain was then divided into 246 regions (Appendix Table 5). DPARSFA in DPABI was employed to generate a FC matrix for the 246 brain regions. Specifically, the Pearson correlation coefficient of the time series of Blood Oxygen Level Dependent (BOLD) signals between each pair of brain regions was calculated to generate the corresponding correlation matrix. Then, Fisher’s z-transformation was applied to convert the correlation matrix to the z-plane for subsequent analysis.

### Statistical Analysis

Statistical analysis was conducted using IBM SPSS Statistics (SPSS version 26.0 for Windows, SPSS, Inc., Chicago, IL, USA). To compare the differences in HAMD scores and HAMD factor scores before and after iTBS treatment, paired-sample *t*-tests were performed after confirming the normality of pre-post treatment change scores in both HAMD scores and HAMD factor scores using Shapiro-Wilk test. The differences in FC across brain regions before and after treatment were also compared using paired-sample *t*-tests, with analysis conducted using the statistical module in DPABI. Results were controlled for by the false discovery rate (FDR) method, with FDR q < 0.05 considered statistically significant. The result figures were generated using MatLab 2023b (MathWorks Inc., Natick, MA, USA.) and Brainnet Viewer (v1.7, http://www.nitrc.org/projects/bnv/) [20]. Significant FC differences were then extracted, and Pearson correlation analysis was performed between baseline values of these FCs and the reduction rate of the scale scores. Additionally, Multiple linear regression (backward method) with general demographic data as control variables will be used to investigate whether the baseline values of these significant FCs could predict reduction rates in HAMD scores and factor scores.

Furthermore, the participants were divided into two subgroups based on whether they responded or not for subgroup analysis to explore the differences in their FCs. Firstly, the independent sample *t*-test was used to compare whether there were differences in FCs between the two subgroups at baseline. Then, an interaction effect analysis was conducted on FCs of the two groups before and after the intervention to explore whether there were differences in the changes of FCs between the two subgroups after the intervention. All were analyzed using DPABI, and the results were corrected for multiple comparisons using FDR. An FDR q < 0.05 was considered significant.

## Results

### Demographic and Clinical Outcomes

A total of 40 participants were recruited for the study. Of these, 5 participants were excluded for not completing the 10-day treatment protocol, 5 participants voluntarily withdrew from the study for personal reasons, and 1 participant was excluded due to failure to meet the MRI data quality assessment criteria. Ultimately, data from 29 participants were included in the analysis (Table 1). Throughout the study, no participants experienced intolerance to iTBS treatment or other adverse reactions.

**Table 1.** **Characteristics of patients between pre-iTBS and post-iTBS**.

Characteristic	pre-iTBS	post-iTBS	*T*	*p*
Age (years)	36.09 ± 12.97			
Male (n, %)	12 (41.38%)			
Education (years)	14.07 ± 4.04			
BMI (kg/m^2^)	23.55 ± 3.67			
HAMD	20.86 ± 4.76	10.45 ± 4.95	10.33	<0.001*
Retardation factor	7.07 ± 1.69	3.66 ± 1.84	8.02	<0.001*
Cognitive impairment factor	3.93 ± 2.00	1.59 ± 1.48	6.19	<0.001*
Anxiety factor	6.10 ± 2.60	3.55 ± 2.10	6.45	<0.001*
Sleep disturbance factor	3.48 ± 1.60	1.62 ± 1.50	5.67	<0.001*

iTBS, intermittent theta-burst stimulation; BMI, body mass index; HAMD, the 17-item Hamilton Depression Rating Scale. The values are represented as the mean ± standard deviation. *p < 0.05, The *p* value is obtained by paired-sample *t*-tests, two-tailed.

Results of the Shapiro-Wilk test indicated that the pre-post iTBS treatment change scores for HAMD scores were normally distributed (*W* = 0.972, *p* = 0.603). Likewise, differences in all HAMD factor scores also demonstrated normality. After 20 sessions of iTBS treatment, participants’ HAMD scores decreased from 20.86 to 10.45, representing a 49.92% reduction, with a response rate of 62.07% and a remission rate of 31.03%, showing a trend toward increase in the severity of depressive symptoms. Correspondingly, these HAMD factor scores were all decrease, with the retardation factor showing the greatest reduction—from 7.07 to 3.66, a decrease of 48.29%.

### rsFC Differences Between Pre- and Post-iTBS Treatment

**Fig. 1.**
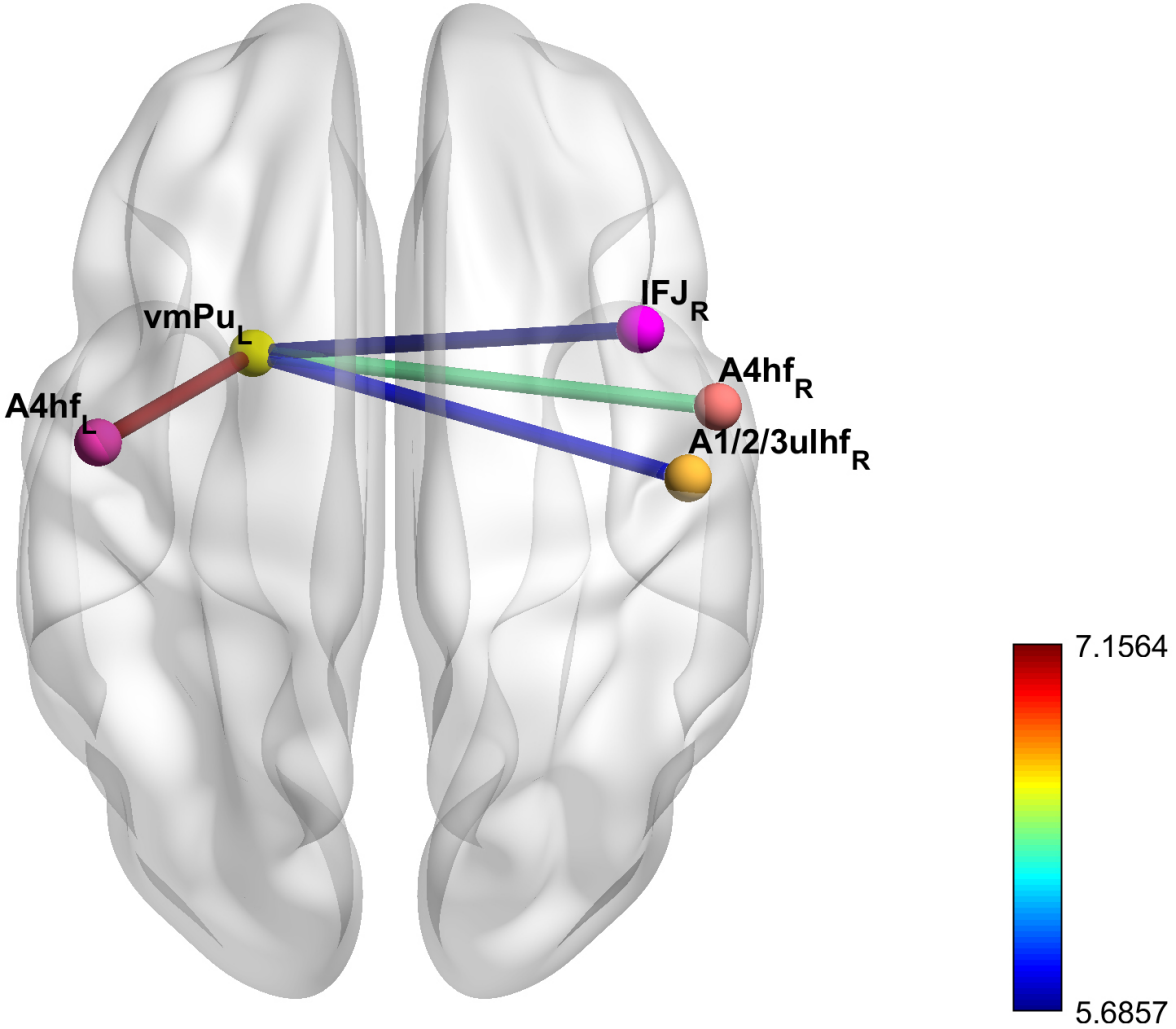
**Functional connectivity with significant differences before and after intermittent theta-burst stimulation (iTBS) treatment**. vmPu, ventromedial putamen; IFJ, inferior frontal junction; A4hf, the precentral gyrus corresponding to Brodmann Areas 4 for the head and face; A1/2/3ulhf, the postcentral gyrus regions corresponding to Brodmann Areas 1, 2, 3 for the upper limb, head, and face. The subscripts at the bottom right of each brain region indicate the hemisphere to which the region belongs, with “L” representing the left hemisphere and “R” representing the right hemisphere. The color of the nodes serves to distinguish different brain regions, while the color of the edges represents the magnitude of the statistical effect size, corresponding to the t-values obtained from the paired sample *t*-test comparing functional connectivity before and after treatment.

The results of the paired-sample *t*-test showed that four pairs of FCs in the FC matrix, constructed using the Brainnetome Atlas, exhibited statistically significant differences before and after iTBS treatment (Fig. 1). These included the FCs between the left ventromedial putamen (vmPu) and the right inferior frontal junction (IFJ), between the vmPu and the bilateral precentral gyrus (PrG) corresponding to Brodmann Areas 4 for the head and face (A4hf), and between the vmPu and the right postcentral gyrus (PoG) corresponding to Brodmann Areas 1, 2, 3 for the upper limb, head, and face (A1/2/3ulhf). All four pairs of FCs were positive at baseline, but after 20 sessions of iTBS treatment, the strength of the positive connectivity decreased.

### Correlation and Regression Analysis of FCWith Clinical Symptoms

**Fig. 2.**
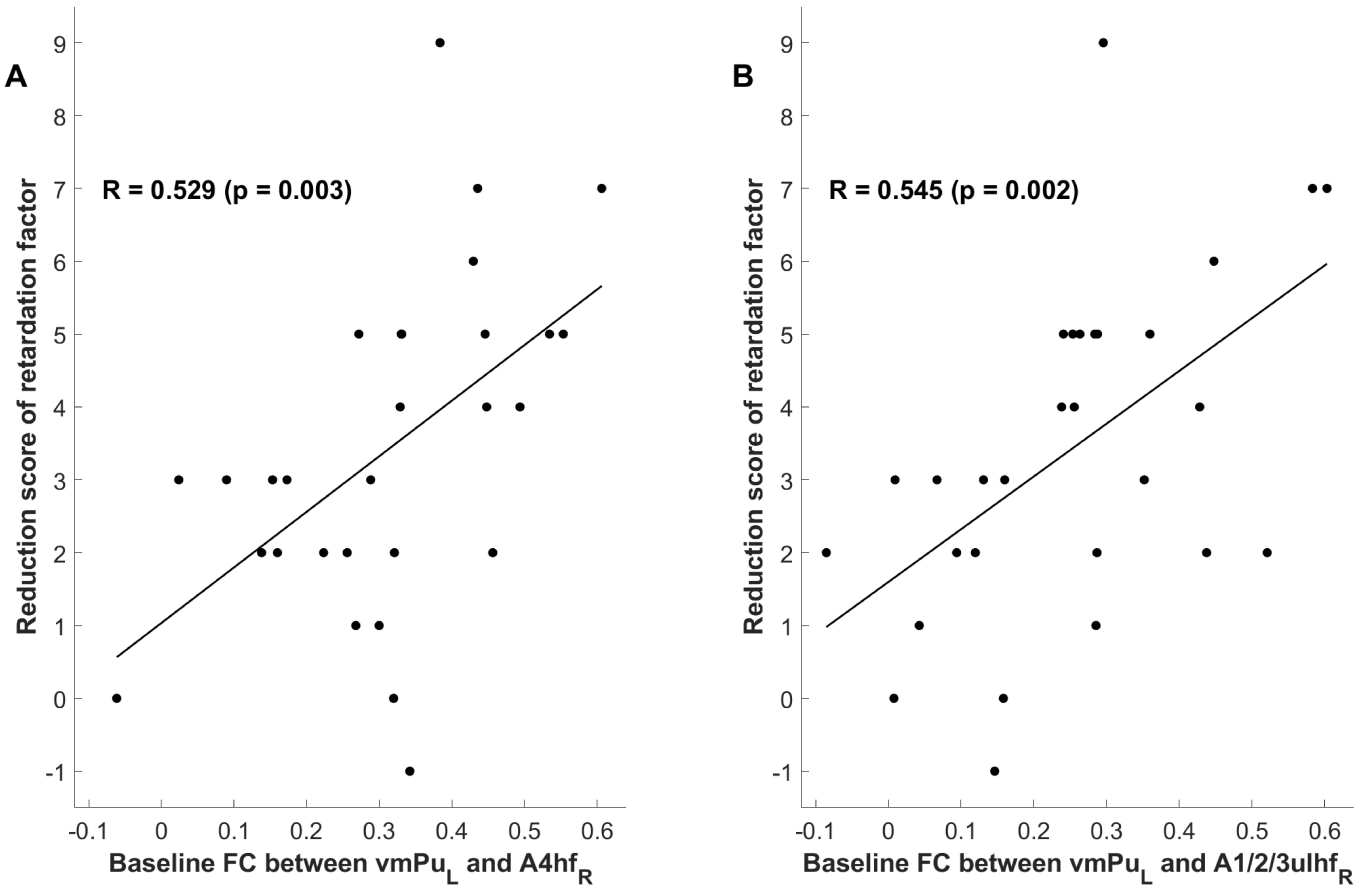
**The correlation between baseline functional connectivity and the reduction score of the retardation factor**. (A) The baseline functional connectivity between the left vmPu and the right A4hf shows a positive correlation with the reduction score of the retardation factor, meaning that higher baseline functional connectivity corresponded to higher reduction score of the retardation factor. (B) The baseline functional connectivity between the left vmPu and the right A1/2/3ulhf also shows a positive correlation with the reduction score of the retardation factor. FC, functional connectivity; vmPu, the ventromedial putamen; A1/2/3ulhf, the postcentral gyrus regions corresponding to Brodmann Areas 1, 2, 3 for the upper limb, head, and face. The subscripts at the bottom right of each brain region indicate the hemisphere to which the region belongs, with “L” representing the left hemisphere and “R” representing the right hemisphere.

Based on the significant FC differences found before, a correlation analysis was performed between their baseline values and clinical symptoms. The results showed no correlation between the reduction rate of HAMD scores and these FCs (Table 2). However, when the reduction scores of each HAMD factor were analyzed, it was found that the baseline FC between the left vmPu and left A4hf was positively correlated with the reduction score of the retardation factor (r = 0.529, *p* = 0.003), and the baseline FC between the left vmPu and right A1/2/3ulhf was also positively correlated with the reduction score of the retardation factor (r = 0.545, *p* = 0.002) (Fig. 2).

**Table 2.** **Correlation between baseline functional connectivity and the reduction rate of HAMD**.

FC		r	*p*
Region1	Region2		
Left vmPu	right IFJ	0.305	0.108
	left A4hf	0.189	0.325
	right A4hf	0.023	0.905
	right A1/2/3ulhf	0.188	0.330

HAMD, the 17-item Hamilton Depression Rating Scale; FC, functional connectivity; vmPu, ventromedial putamen; IFJ, inferior frontal junction; A4hf, the precentral gyrus corresponding to Brodmann Areas 4 for the head and face; A1/2/3ulhf, the postcentral gyrus regions corresponding to Brodmann Areas 1, 2, 3 for the upper limb, head, and face.

A Multiple linear regression analysis was conducted between the baseline values of these significant FCs and depression symptoms. The baseline FC values were modeled in a regression analysis incorporating all control variables, then the final model showed that after excluding the confounding effect of general demographic data, the baseline FC between right IFJ and left vmPu could significantly affect the HAMD reduction rate (*p* = 0.024), and the model explained 34.9% of the variance of the reduction rate (Table 3). Similarly, when these FCs were used to build linear regression models for the reduction rates of each HAMD factor, it can also be found that after excluding the confounding interference of general demographic data, the baseline FC between the left vmPu and the left A4hf can significantly predict the reduction rate of the retardation factor (*p* = 0.014), and the model explains 27.8% of the variance of the reduction rate (Table 4).

**Table 3.** **Predictor variables of the reduction rate of HAMD**.

	Unstandardized coefficients		Standardized coefficients	*T*	*p*	Adjusted *R*^2^
	B	Standard Error	Beta			
Intercept	–0.158	0.349		–0.453	0.655	0.349
FC between vmPu_L and IFJ_R	0.563	0.234	0.435	2.411	0.024*	
Age	0.006	0.003	0.317	1.737	0.095	
Education	–0.005	0.011	–0.089	–0.463	0.648	
BMI	0.016	0.012	0.256	1.360	0.186	

Dependent variable: Reduction rate of HAMD. HAMD, the 17-item Hamilton Depression Rating Scale; FC, functional connectivity; vmPu_L, the left ventromedial putamen; IFJ_R, the right inferior frontal junction; BMI, body mass index. * *p* < 0.05, two-tailed.

**Table 4.** **Predictor variables of the reduction rate of Retardation factor**.

	Unstandardized coefficients		Standardized coefficients	*T*	*p*	Adjusted *R*^2^
	B	Standard Error	Beta			
Intercept	–0.179	0.484		–0.370	0.715	0.278
FC between vmPu_L and A4hf_L	0.924	0.350	0.520	2.637	0.014*	
Age	0.006	0.004	0.296	1.544	0.136	
Education	–0.002	0.014	–0.023	–0.119	0.906	
BMI	0.006	0.016	0.079	0.383	0.705	

Dependent variable: Reduction rate of Retardation factor. FC, functional connectivity; vmPu_L, the left ventromedial putamen; A4hf_L, the left precentral gyrus corresponding to Brodmann Areas 4 for the head and face; BMI, body mass index. * *p* < 0.05, two-tailed.

### Subgroup Analysis

Participants were divided into two subgroups based on the HAMD reduction rate, and there were no differences in general demographics such as age and gender between the two subgroups (**Supplementary Table 1**). By comparing the baseline FCs in the FC matrix constructed from the Brainnnetome Atlas, it was found that, after multiple comparison correction using FDR, there was no significant difference (**Supplementary Fig. 1**) in the baseline FC between the two subgroups. The results before correction were shown in **Supplementary Fig. 1**.

In the interaction analysis of groups and interventions, no FC with significant differences (**Supplementary Fig. 2**) in changes of FC before and after the intervention was found between the two subgroups. The results of the uncorrected interaction effect are shown in **Supplementary Fig. 2**.

## Discussion

This study finds that, after left DLPFC iTBS treatment in MDD patients, there are significant reductions in HAMD scores, as well as positive FC between the left vmPu and right IFJ, bilateral PrG, and left PoG. Additionally, the vmPu FC with the left PrG and the right PoG was positively correlated with the reduction in retardation factor scores and was able to predict improvements in retardation symptoms.

As shown in the results of the network meta-analysis on Theta burst stimulation in the treatment of depression, iTBS of the left DLPFC has a favorable risk-benefit balance because of its high efficiency, better acceptability and safety profiles [21]. In this study, not bad response rate (62.07%) and remission rate (31.03%) of iTBS treatment for MDD were reported. This promising clinical improvement is precisely a guarantee for the reliability of the subsequent FC analysis results and also a prerequisite for judging whether the predictors discovered in this study have practical significance for clinical application. Furthermore, compared with standard iTBS, accelerated iTBS can significantly shorten the overall treatment duration and achieve a faster antidepressant effect [10], which clarifies our subsequent research direction. It is worth noting that the meta-analysis by Cai *et al*. [22]. indicates that the results of accelerating iTBS in the treatment of different subtypes of depression, such as MDD and bipolar disorder, are inconsistent in terms of efficacy and safety. This suggests the importance of adhering to the classification of subtypes and populations as in current studies for follow-up research.

Our analysis of FC identified the vmPu as a prominent node in the results, serving as a common node for the four pairs of FCs that changed after iTBS intervention. The vmPu plays an important role in the reward circuit [23], primarily linking reward anticipation. A study on MDD patients have shown that the activation level of the putamen during reward processing is often lower than that of healthy individuals [24]. In addition, it was noted that decreased FC between the right putamen and the right orbitofrontal cortex, as well as between the left putamen and the subcortex of the corpus callosum, has been found to predict changes in the Beck Depression Inventory (BDI) during the reward anticipation phase. Moreover, abnormalities in the volume and shape of the putamen were found in patients with untreated first-episode MDD [25], and this was confirmed in a neuroimaging meta-analysis [26]. Furthermore, this meta-analysis also identified diminished putamen function in patients with MDD [26]. This underscores the necessity of further investigating the potential role of the putamen in MDD, such as by examining the correlation between anhedonia severity and aberrant neural activity in response to emotional stimuli [27]. In our correlation analysis, two of the four pairs of vmPu FC are associated with changes in the retardation factor scores of depressive symptoms, which is inconsistent with Harlé *et al*.’s study [28]. They found that clinical measurements were not significantly associated with ventromedial prefrontal cortex (vmPFC) or putamen activation. The differences in the study population are obvious, which is an important potential reason that cannot be ignored leading to the inconsistency of the relevant analysis results.

A4hf and A1/2/3ulhf belong to the PrG and the PoG, respectively, but are both part of the somatomotor network (SMN) in the Yeo *et al*.’s 7 network [29]. They are both involved in functions such as facial expression, speech, and head control [19]. Their functional changes are believed to be closely associated with core symptoms of depression, such as psychomotor retardation [30]. Moreover, FC abnormalities in SMN have also been shown to be related to psychomotor retardation by previous studies [31, 32]. Our study found that the FC between the vmPu and A4hf, as well as between the vmPu and A1/2/3ulhf, was weakened after treatment, and the baseline FC between them was correlated with changes in the retardation factor. Although a previous study suggested that the FC between the putamen and SMN was associated with obsessive-compulsive symptoms [33], there is limited evidence from other relevant studies, and the results regarding FC between the putamen and SMN remain unclear. We hypothesized that there is a regulatory effect involving psychomotor retardation between the vmPu, as part of the reward circuit, and the SMN. Abnormal FC between these regions may reflect the retardation symptoms in speech and movement observed in MDD patients [34]. Therefore, a correlation was detected between their baseline FC and changes in the retardation factor in this study. Furthermore, the results also showed that the baseline FC between the left A4hf and the left vmPu could predict changes in the retardation factor in MDD patients. This may provide some neuroimaging evidence for the potential of iTBS intervention to improve core symptoms of MDD, particularly symptoms like reduced interest, as well as movement and speech retardation.

The IFJ is considered part of the cognitive control network, and the results of a meta-analysis support this hypothesis, finding significant co-activation with the putamen [35]. This study found a decrease in FC between vmPu and IFJ after treatment, meaning that these two brain regions deviated from the co-activation pattern following the intervention. It can be inferred that, even after receiving the intervention and showing symptom improvement, differences in the activation of these two brain regions may persist between depressed subjects and healthy individuals. Of course, the possibility of a false positive cannot be ruled out. Therefore, the resulting reliability of the reduction rate of baseline FC in predicting HAMD between IJF and vmPu in the regression analysis seems to be questionable.

In the subgroup analysis, no significant difference in baseline FC between responders and non-responders was found. If we looked at the uncorrected results, we found potential differences in FC in the cingulate, parietal lobe, and insula, which were consistent with the predictors of response prediction in previous studies [36, 37]. Furthermore, the higher connectivity of the response group compared to the non-response group was consistent with the results of the correlation analysis, that is, a high level of baseline FC was associated with a higher reduction rate of the HAMD factors score. However, Ge *et al*. [37] found that the non-response group had higher connectivity in the default mode network than the response group, which was contrary to our results. But the results of both were limited to a smaller sample size. Moreover, we explored whether there were significant differences in the changes of FC between the two subgroups after the intervention. Our result was negative, as no significant changes in FC survived FDR correction. Only potential changes in FC between the orbital gyrus (A14m) and cingulate gyrus (A23v), as well as dorsal caudate (dCa) were found, which were manifested as a greater reduction in these FCs in the response group compared to the non-response group after the intervention. Perhaps it can be considered that greater changes in FC in brain regions such as the cingulate gyrus, which are related to emotion processing, have contributed to more improvements in depressive symptoms [38], thereby reflecting this difference.

It is important to acknowledge several limitations in this study. Firstly, the sample size is relatively small, and future research could increase the sample size to reduce the likelihood of random findings. Additionally, this study did not include a control group and only compared the treatment effects in MDD patients before and after intervention. Future studies could consider including a control group of healthy individuals for comparison, which would provide more depth to the results. Moreover, FC between left vmPu and right IFJ is only a partial predictor. In subsequent studies, it is necessary to combine other biomarkers or clinical characteristics to construct a more complete prediction model.

## Conclusion

Overall, iTBS intervention targeting the left DLPFC has shown promising therapeutic effects in treating depression. During exploratory analysis, reductions in positive FC between the left vmPu and right IFJ, bilateral PrG, and left PoG were detected. Furthermore, the predictive effects of FC between baseline vmPu and IFJ as well as PrG on the improvement of depressive symptoms and retardation symptoms were discovered respectively. Subsequent studies should expand the sample size, optimize the experimental design, and incorporate additional biomarkers or clinical indicators to construct a more comprehensive prediction model.

## Availability of Data and Materials

The datasets generated and/or analyzed during the cur-rent study are available from the corresponding author on reasonable request.

## Supplementary Material

Supplementary material associated with this article can be found, in the online version, at https://doi.org/10.62641/aep.v53i6.1983.

## Appendix

See Table 5.

**Table 5.** **BNA_subregions**.

Lobe	Gyrus	Left and right hemisphere	Label ID.L	Label ID.R	Anatomical and modified Cyto-architectonic descriptions	lh.MNI(X,Y,Z)	rh.MNI(X,Y,Z)
Frontal Lobe	SFG, Superior Frontal Gyrus	SFG_L(R)_7_1	1	2	*A8m, medial area 8*	–5, 15, 54	7, 16, 54
		SFG_L(R)_7_2	3	4	*A8dl, dorsolateral area 8*	–18, 24, 53	22, 26, 51
		SFG_L(R)_7_3	5	6	*A9l, lateral area 9*	–11, 49, 40	13, 48, 40
		SFG_L(R)_7_4	7	8	*A6dl, dorsolateral area 6*	–18, –1, 65	20, 4, 64
		SFG_L(R)_7_5	9	10	*A6m, medial area 6*	–6, –5, 58	7, –4, 60
		SFG_L(R)_7_6	11	12	*A9m,medial area 9*	–5, 36, 38	6, 38, 35
		SFG_L(R)_7_7	13	14	*A10m, medial area 10*	–8, 56, 15	8, 58, 13
	MFG, Middle Frontal Gyrus	MFG_L(R)_7_1	15	16	*A9/46d, dorsal area 9/46*	–27, 43, 31	30, 37, 36
		MFG_L(R)_7_2	17	18	*IFJ, inferior frontal junction*	–42, 13, 36	42, 11, 39
		MFG_L(R)_7_3	19	20	*A46, area 46*	–28, 56, 12	28, 55, 17
		MFG_L(R)_7_4	21	22	*A9/46v, ventral area 9/46*	–41, 41, 16	42, 44, 14
		MFG_L(R)_7_5	23	24	*A8vl, ventrolateral area 8*	–33, 23, 45	42, 27, 39
		MFG_L(R)_7_6	25	26	*A6vl, ventrolateral area 6*	–32, 4, 55	34, 8, 54
		MFG_L(R)_7_7	27	28	*A10l, lateral area10*	–26, 60, –6	25, 61, –4
	IFG, Inferior Frontal Gyrus	IFG_L(R)_6_1	29	30	*A44d, dorsal area 44*	–46, 13, 24	45, 16, 25
		IFG_L(R)_6_2	31	32	*IFS, inferior frontal sulcus*	–47, 32, 14	48, 35, 13
		IFG_L(R)_6_3	33	34	*A45c, caudal area 45*	–53, 23, 11	54, 24, 12
		IFG_L(R)_6_4	35	36	*A45r, rostral area 45*	–49, 36, –3	51, 36, –1
		IFG_L(R)_6_5	37	38	*A44op, opercular area 44*	–39, 23, 4	42, 22, 3
		IFG_L(R)_6_6	39	40	*A44v, ventral area 44*	–52, 13, 6	54, 14, 11
	OrG, Orbital Gyrus	OrG_L(R)_6_1	41	42	*A14m, medial area 14*	–7, 54, –7	6, 47, –7
		OrG_L(R)_6_2	43	44	*A12/47o, orbital area 12/47*	–36, 33, –16	40, 39, –14
		OrG_L(R)_6_3	45	46	*A11l, lateral area 11*	–23, 38, –18	23, 36, –18
		OrG_L(R)_6_4	47	48	*A11m, medial area 11*	–6, 52, –19	6, 57, –16
		OrG_L(R)_6_5	49	50	*A13, area 13*	–10, 18, –19	9, 20, –19
		OrG_L(R)_6_6	51	52	*A12/47l, lateral area 12/47*	–41, 32, –9	42, 31, –9
	PrG, Precentral Gyrus	PrG_L(R)_6_1	53	54	*A4hf, area 4 (head and face region)*	–49, –8, 39	55, –2, 33
		PrG_L(R)_6_2	55	56	*A6cdl, caudal dorsolateral area 6*	–32, –9, 58	33, –7, 57
		PrG_L(R)_6_3	57	58	*A4ul, area 4 (upper limb region)*	–26, –25, 63	34, –19, 59
		PrG_L(R)_6_4	59	60	*A4t, area 4 (trunk region)*	–13, –20, 73	15, –22, 71
		PrG_L(R)_6_5	61	62	*A4tl, area 4 (tongue and larynx region)*	–52, 0, 8	54, 4, 9
		PrG_L(R)_6_6	63	64	*A6cvl, caudal ventrolateral area 6*	–49, 5, 30	51, 7, 30
	PCL, Paracentral Lobule	PCL_L(R)_2_1	65	66	*A1/2/3ll, area1/2/3 (lower limb region)*	–8, –38, 58	10, –34, 54
		PCL_L(R)_2_2	67	68	*A4ll, area 4, (lower limb region)*	–4, –23, 61	5, –21, 61
Temporal Lobe	STG, Superior Temporal Gyrus	STG_L(R)_6_1	69	70	*A38m, medial area 38*	–32, 14, –34	31, 15, –34
		STG_L(R)_6_2	71	72	*A41/42, area 41/42*	–54, –32, 12	54, –24, 11
		STG_L(R)_6_3	73	74	*TE1.0 and TE1.2*	–50, –11, 1	51, –4, –1
		STG_L(R)_6_4	75	76	*A22c, caudal area 22*	–62, –33, 7	66, –20, 6
		STG_L(R)_6_5	77	78	*A38l, lateral area 38*	–45, 11, –20	47, 12, –20
		STG_L(R)_6_6	79	80	*A22r, rostral area 22*	–55, –3, –10	56, –12, –5
	MTG, Middle Temporal Gyrus	MTG_L(R)_4_1	81	82	*A21c, caudal area 21*	–65, –30, –12	65, –29, –13
		MTG_L(R)_4_2	83	84	*A21r, rostral area 21*	–53, 2, –30	51, 6, –32
		MTG_L(R)_4_3	85	86	*A37dl, dorsolateral area37*	–59, –58, 4	60, –53, 3
		MTG_L(R)_4_4	87	88	*aSTS, anterior superior temporal sulcus*	–58, –20, –9	58, –16, –10
	ITG, Inferior Temporal Gyrus	ITG_L(R)_7_1	89	90	*A20iv, intermediate ventral area 20*	–45, –26, –27	46, –14, –33
		ITG_L(R)_7_2	91	92	*A37elv, extreme lateroventral area37*	–51, –57, –15	53, –52, –18
		ITG_L(R)_7_3	93	94	*A20r, rostral area 20*	–43, –2, –41	40, 0, –43
		ITG_L(R)_7_4	95	96	*A20il, intermediate lateral area 20*	–56, –16, –28	55, –11, –32
		ITG_L(R)_7_5	97	98	*A37vl, ventrolateral area 37*	–55, –60, –6	54, –57, –8
		ITG_L(R)_7_6	99	100	*A20cl, caudolateral of area 20*	–59, –42, –16	61, –40, –17
		ITG_L(R)_7_7	101	102	*A20cv, caudoventral of area 20*	–55, –31, –27	54, –31, –26
	FuG, Fusiform Gyrus	FuG_L(R)_3_1	103	104	*A20rv, rostroventral area 20*	–33, –16, –32	33, –15, –34
		FuG_L(R)_3_2	105	106	*A37mv, medioventral area37*	–31, –64, –14	31, –62, –14
		FuG_L(R)_3_3	107	108	*A37lv, lateroventral area37*	–42, –51, –17	43, –49, –19
	PhG, Parahippocampal Gyrus	PhG_L(R)_6_1	109	110	*A35/36r, rostral area 35/36*	–27, –7, –34	28, –8, –33
		PhG_L(R)_6_2	111	112	*A35/36c, caudal area 35/36*	–25, –25, –26	26, –23, –27
		PhG_L(R)_6_3	113	114	*TL, area TL (lateral PPHC, posterior parahippocampal gyrus)*	–28, –32, –18	30, –30, –18
		PhG_L(R)_6_4	115	116	*A28/34, area 28/34 (EC, entorhinal cortex)*	–19, –12, –30	19, –10, –30
		PhG_L(R)_6_5	117	118	*TI, area TI (temporal agranular insular cortex)*	–23, 2, –32	22, 1, –36
		PhG_L(R)_6_6	119	120	*TH, area TH (medial PPHC)*	–17, –39, –10	19, –36, –11
	pSTS, posterior Superior Temporal Sulcus	pSTS_L(R)_2_1	121	122	*rpSTS, rostroposterior superior temporal sulcus*	–54, –40, 4	53, –37, 3
		pSTS_L(R)_2_2	123	124	*cpSTS, caudoposterior superior temporal sulcus*	–52, –50, 11	57, –40, 12
Parietal Lobe	SPL, Superior Parietal Lobule	SPL_L(R)_5_1	125	126	*A7r, rostral area 7*	–16, –60, 63	19, –57, 65
		SPL_L(R)_5_2	127	128	*A7c, caudal area 7*	–15, –71, 52	19, –69, 54
		SPL_L(R)_5_3	129	130	*A5l, lateral area 5*	–33, –47, 50	35, –42, 54
		SPL_L(R)_5_4	131	132	*A7pc, postcentral area 7*	–22, –47, 65	23, –43, 67
		SPL_L(R)_5_5	133	134	*A7ip, intraparietal area 7 (hIP3)*	–27, –59, 54	31, –54, 53
	IPL, Inferior Parietal Lobule	IPL_L(R)_6_1	135	136	*A39c, caudal area 39 (PGp)*	–34, –80, 29	45, –71, 20
		IPL_L(R)_6_2	137	138	*A39rd, rostrodorsal area 39 (Hip3)*	–38, –61, 46	39, –65, 44
		IPL_L(R)_6_3	139	140	*A40rd, rostrodorsal area 40 (PFt)*	–51, –33, 42	47, –35, 45
		IPL_L(R)_6_4	141	142	*A40c, caudal area 40 (PFm)*	–56, –49, 38	57, –44, 38
		IPL_L(R)_6_5	143	144	*A39rv, rostroventral area 39 (PGa)*	–47, –65, 26	53, –54, 25
		IPL_L(R)_6_6	145	146	*A40rv, rostroventral area 40 (PFop)*	–53, –31, 23	55, –26, 26
	Pcun, Precuneus	PCun_L(R)_4_1	147	148	*A7m, medial area 7 (PEp)*	–5, –63, 51	6, –65, 51
		PCun_L(R)_4_2	149	150	*A5m, medial area 5 (PEm)*	–8, –47, 57	7, –47, 58
		PCun_L(R)_4_3	151	152	*dmPOS, dorsomedial parietooccipital sulcus (PEr)*	–12, –67, 25	16, –64, 25
		PCun_L(R)_4_4	153	154	*A31, area 31 (Lc1)*	–6, –55, 34	6, –54, 35
	PoG, Postcentral Gyrus	PoG_L(R)_4_1	155	156	*A1/2/3ulhf, area 1/2/3 (upper limb, head and face region)*	–50, –16, 43	50, –14, 44
		PoG_L(R)_4_2	157	158	*A1/2/3tonIa, area 1/2/3 (tongue and larynx region)*	–56, –14, 16	56, –10, 15
		PoG_L(R)_4_3	159	160	*A2, area 2*	–46, –30, 50	48, –24, 48
		PoG_L(R)_4_4	161	162	*A1/2/3tru, area1/2/3 (trunk region)*	–21, –35, 68	20, –33, 69
Insular Lobe	INS, Insular Gyrus	INS_L(R)_6_1	163	164	*G, hypergranular insula*	–36, –20, 10	37, –18, 8
		INS_L(R)_6_2	165	166	*vIa, ventral agranular insula*	–32, 14, –13	33, 14, –13
		INS_L(R)_6_3	167	168	*dIa, dorsal agranular insula*	–34, 18, 1	36, 18, 1
		INS_L(R)_6_4	169	170	*vId/vIg, ventral dysgranular and granular insula*	–38, –4, –9	39, –2, –9
		INS_L(R)_6_5	171	172	*dIg, dorsal granular insula*	–38, –8, 8	39, –7, 8
		INS_L(R)_6_6	173	174	*dId, dorsal dysgranular insula*	–38, 5, 5	38, 5, 5
Limbic Lobe	CG, Cingulate Gyrus	CG_L(R)_7_1	175	176	*A23d, dorsal area 23*	–4, –39, 31	4, –37, 32
		CG_L(R)_7_2	177	178	*A24rv, rostroventral area 24*	–3, 8, 25	5, 22, 12
		CG_L(R)_7_3	179	180	*A32p, pregenual area 32*	–6, 34, 21	5, 28, 27
		CG_L(R)_7_4	181	182	*A23v, ventral area 23*	–8, –47, 10	9, –44, 11
		CG_L(R)_7_5	183	184	*A24cd, caudodorsal area 24*	–5, 7, 37	4, 6, 38
		CG_L(R)_7_6	185	186	*A23c, caudal area 23*	–7, –23, 41	6, –20, 40
		CG_L(R)_7_7	187	188	*A32sg, subgenual area 32*	–4, 39, –2	5, 41, 6
Occipital Lobe	MVOcC*, *MedioVentral Occipital Cortex	MVOcC _L(R)_5_1	189	190	*cLinG, caudal lingual gyrus*	–11, –82, –11	10, –85, –9
		MVOcC _L(R)_5_2	191	192	*rCunG, rostral cuneus gyrus*	–5, –81, 10	7, –76, 11
		MVOcC _L(R)_5_3	193	194	*cCunG, caudal cuneus gyrus*	–6, –94, 1	8, –90, 12
		MVOcC _L(R)_5_4	195	196	*rLinG, rostral lingual gyrus*	–17, –60, –6	18, –60, –7
		MVOcC _L(R)_5_5	197	198	*vmPOS, ventromedial parietooccipital sulcus*	–13, –68, 12	15, –63, 12
	LOcC, lateral Occipital Cortex	LOcC_L(R)_4_1	199	200	*mOccG, middle occipital gyrus*	–31, –89, 11	34, –86, 11
		LOcC _L(R)_4_2	201	202	*V5/MT+, area V5/MT+*	–46, –74, 3	48, –70, –1
		LOcC _L(R)_4_3	203	204	*OPC, occipital polar cortex*	–18, –99, 2	22, –97, 4
		LOcC_L(R)_4_4	205	206	*iOccG, inferior occipital gyrus*	–30, –88, –12	32, –85, –12
		LOcC _L(R)_2_1	207	208	*msOccG, medial superior occipital gyrus*	–11, –88, 31	16, –85, 34
		LOcC _L(R)_2_2	209	210	*lsOccG, lateral superior occipital gyrus*	–22, –77, 36	29, –75, 36
Subcortical Nuclei	Amyg, Amygdala	Amyg_L(R)_2_1	211	212	*mAmyg, medial amygdala*	–19, –2, –20	19, –2, –19
		Amyg_L(R)_2_2	213	214	*lAmyg, lateral amygdala*	–27, –4, –20	28, –3, –20
	Hipp, Hippocampus	Hipp_L(R)_2_1	215	216	*rHipp, rostral hippocampus*	–22, –14, –19	22, –12, –20
		Hipp_L(R)_2_2	217	218	*cHipp, caudal hippocampus*	–28, –30, –10	29, –27, –10
	BG, Basal Ganglia	BG_L(R)_6_1	219	220	*vCa, ventral caudate*	–12, 14, 0	15, 14, –2
		BG_L(R)_6_2	221	222	*GP, globus pallidus*	–22, –2, 4	22, –2, 3
		BG_L(R)_6_3	223	224	*NAC, nucleus accumbens*	–17, 3, –9	15, 8, –9
		BG_L(R)_6_4	225	226	*vmPu, ventromedial putamen*	–23, 7, –4	22, 8, –1
		BG_L(R)_6_5	227	228	*dCa, dorsal caudate*	–14, 2, 16	14, 5, 14
		BG_L(R)_6_6	229	230	*dlPu, dorsolateral putamen*	–28, –5, 2	29, –3, 1
	Tha, Thalamus	Tha_L(R)_8_1	231	232	*mPFtha, medial pre-frontal thalamus*	–7, –12, 5	7, –11, 6
		Tha_L(R)_8_2	233	234	*mPMtha, pre-motor thalamus*	–18, –13, 3	12, –14, 1
		Tha_L(R)_8_3	235	236	*Stha, sensory thalamus*	–18, –23, 4	18, –22, 3
		Tha_L(R)_8_4	237	238	*rTtha, rostral temporal thalamus*	–7, –14, 7	3, –13, 5
		Tha_L(R)_8_5	239	240	*PPtha, posterior parietal thalamus*	–16, –24, 6	15, –25, 6
		Tha_L(R)_8_6	241	242	*Otha, occipital thalamus*	–15, –28, 4	13, –27, 8
		Tha_L(R)_8_7	243	244	*cTtha, caudal temporal thalamus*	–12, –22, 13	10, –14, 14
		Tha_L(R)_8_8	245	246	*lPFtha, lateral pre-frontal thalamus*	–11, –14, 2	13, –16, 7
