# Supplementary material for: Associations Between Left DLPFC iTBS-induced Functional Connectivity Changes and Depressive Symptoms: An Exploratory Study
Source: Actas Esp Psiquiatr. 2025 Dec 17;53(6):1237–51. doi: 10.62641/aep.v53i6.1983 (PMC12728542; doi:10.62641/aep.v53i6.1983)
Supplement: Supplementary file 1 [file ActEsp-53-6-1237-1251-s1.zip › Supplementary material.docx]

**Supplementary Table 1.** Characteristics of patients between response group and non-response group.

| Characteristic | Response (n=18) | Non-response (n=11) | *T/χ*^2^ | *p* |
| --- | --- | --- | --- | --- |
| Age(years) | 38.99±13.08 | 31.37±11.83 | 1.583^a^ | 0.125 |
| Male (n, %) | 7 (38.89%) | 5 (45.45%) | 0.121^b^ | 0.728 |
| Education (years) | 13.78±4.82 | 14.55±2.42 | -0.489^a^ | 0.629 |
| BMI (kg/m^2^) | 24.07±3.75 | 22.71±3.54 | 0.972^a^ | 0.339 |

BMI, body mass index; HAMD, the 17-item Hamilton Depression Rating Scale. a, *T* of the two-sample t-test; b, *χ*^2^ of the chi-square test; The values are represented as the mean ± standard deviation. The p value is obtained by independent sample t-tests, two-tailed.


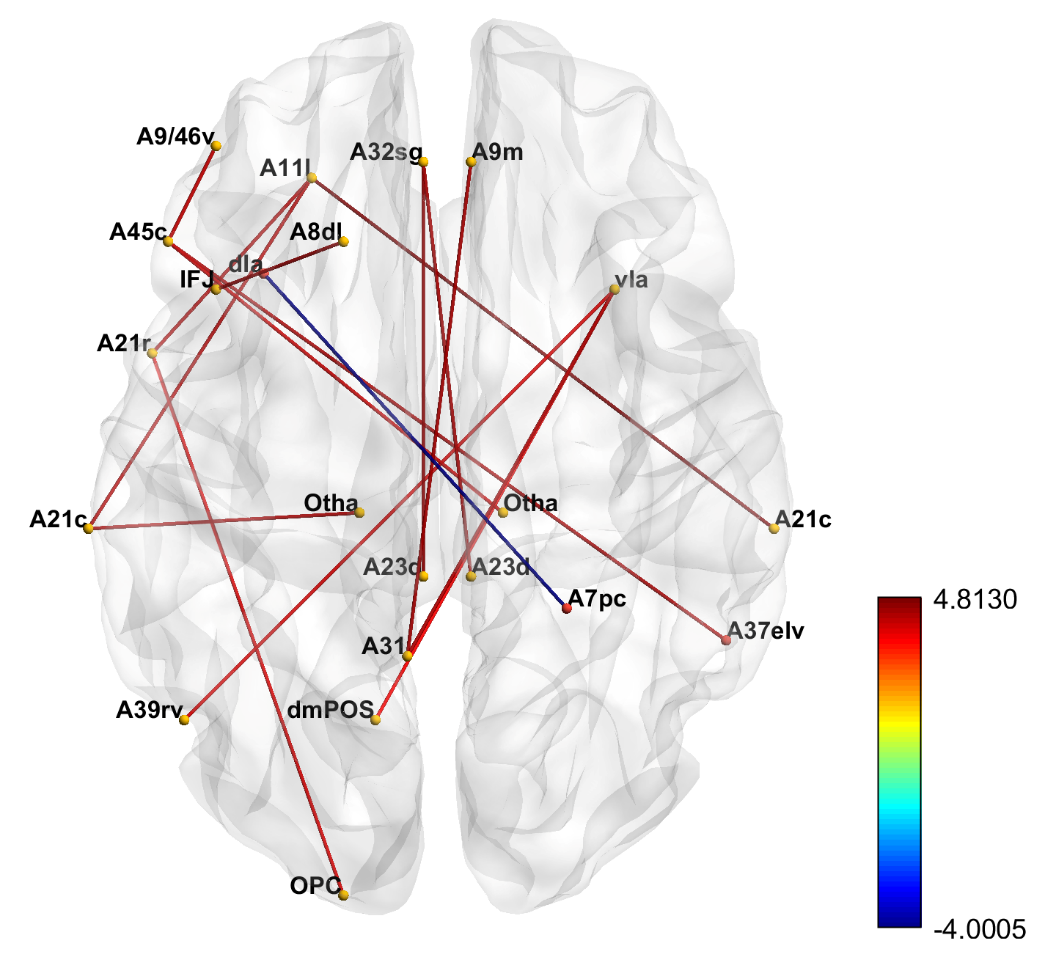


**Supplementary Fig. 1.** Differences of baseline functional connectivity between the response group and the non-response group (uncorrected, p<0.001)

The abbreviations of node names are provided in the Appendix BNA_subregions.xlsx. The color of the nodes serves to distinguish different brain regions, while the color of the edges represents the magnitude of the statistical effect size, corresponding to the t-values obtained from the independent sample t-test comparing baseline functional connectivity.


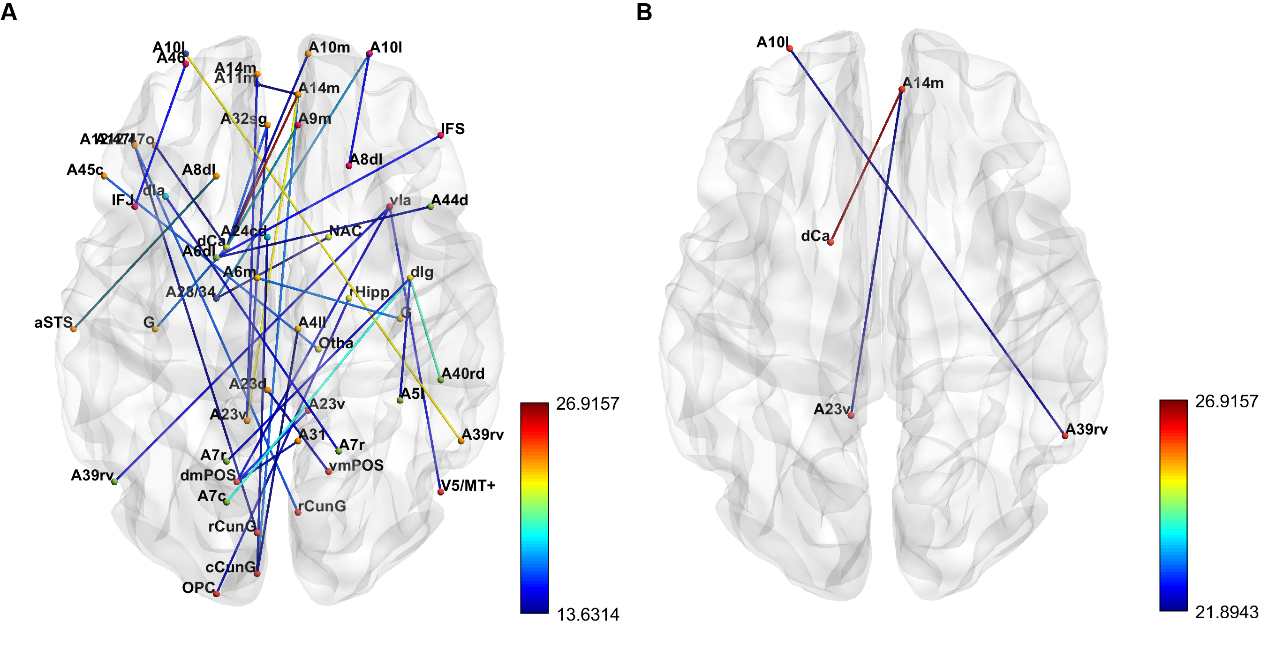


**Supplementary Fig. 2.** The interaction effect between subgroup and intervention (uncorrected).

A, uncorrected, p<0.001;

B, uncorrected, p<0.0001.

The abbreviations of node names are provided in the Appendix BNA_subregions.xlsx. The color of the nodes serves to distinguish different brain regions, while the color of the edges represents the magnitude of the statistical effect size, corresponding to the F-values obtained from interaction effect analysis.
