# Supplementary figures and images for: Associations Between Left DLPFC iTBS-induced Functional Connectivity Changes and Depressive Symptoms: An Exploratory Study
Source: Actas Esp Psiquiatr. 2025 Dec 17;53(6):1237–51. doi: 10.62641/aep.v53i6.1983 (PMC12728542; doi:10.62641/aep.v53i6.1983)

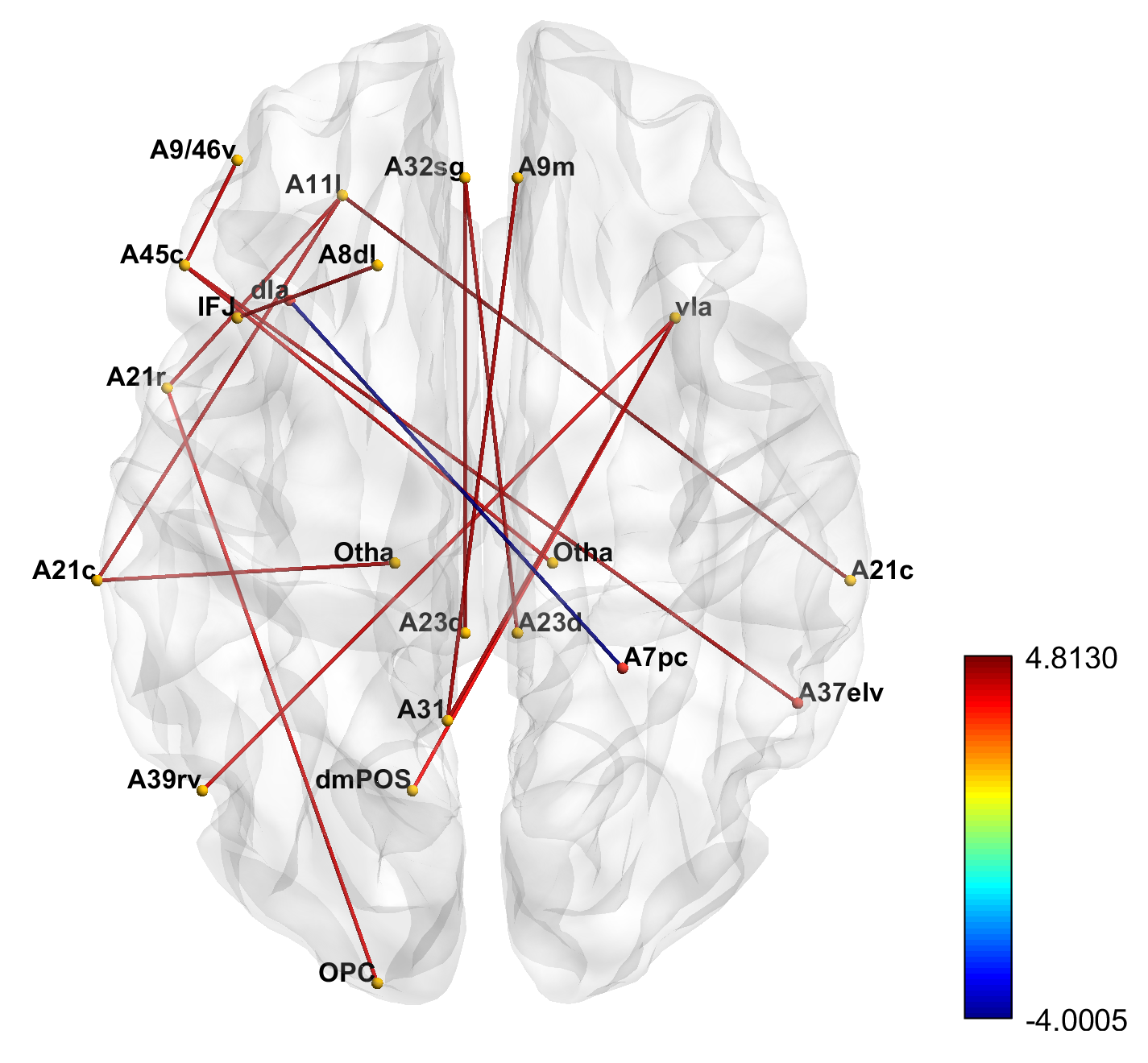

Supplement: Supplementary file 1 [file ActEsp-53-6-1237-1251-s1.zip › Supplementary Fig. 1.tif]

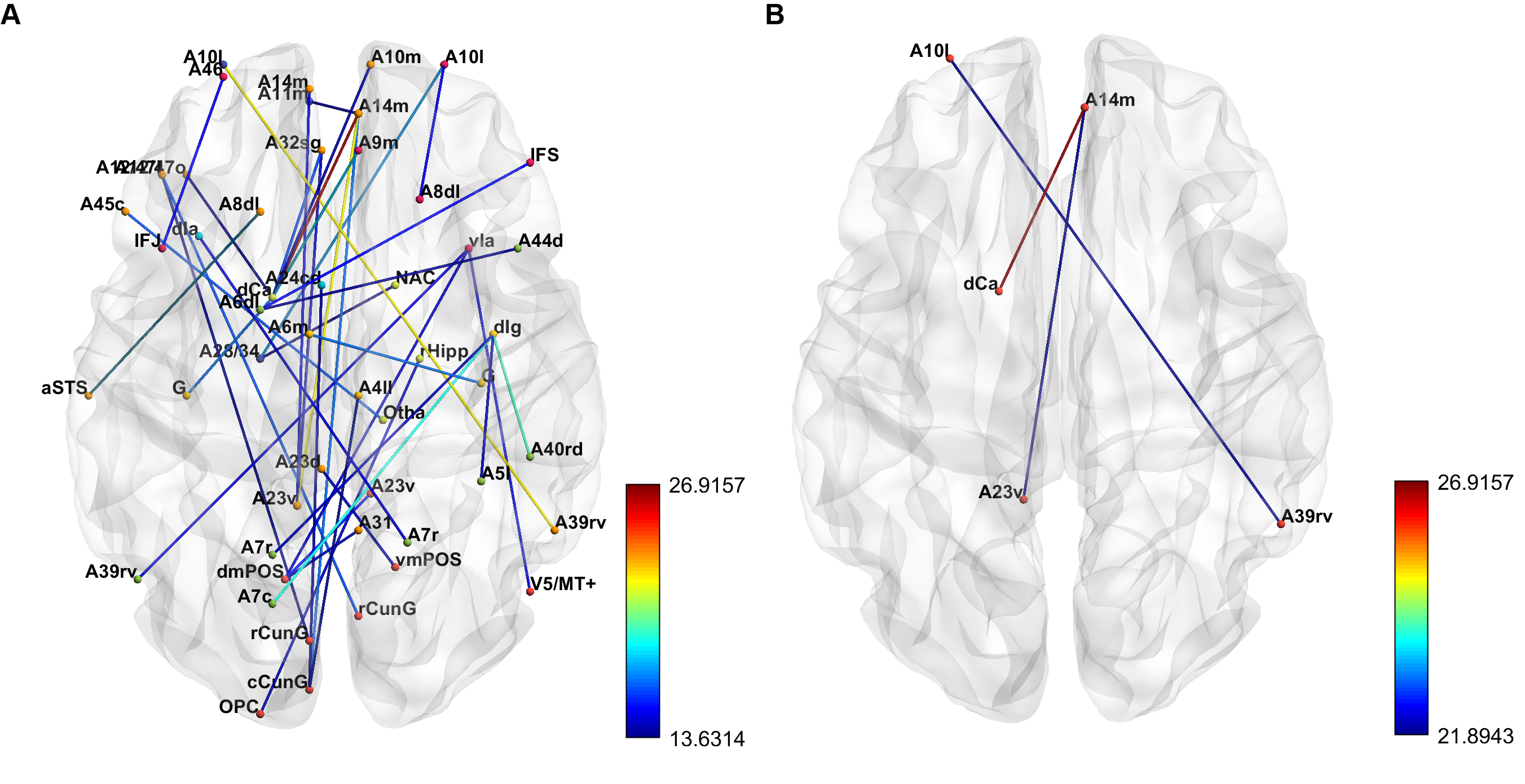

Supplement: Supplementary file 1 [file ActEsp-53-6-1237-1251-s1.zip › Supplementary Fig. 2.tif]
